# Optimizing microwave-assisted extraction of ursolic acid from apple pomace using response surface methodology

**DOI:** 10.3389/fnut.2025.1604863

**Published:** 2025-06-06

**Authors:** Xiyun Sun, Wenjian Zhang, Xianjun Meng, Sajad Shokri

**Affiliations:** ^1^College of Food Science, Shenyang Agricultural University, Shenyang, China; ^2^Department of Chemical Engineering, University of Bath, Bath, United Kingdom

**Keywords:** food waste valorization, ursolic acid, apple pomace, response surface methodology, bioactive compounds

## Abstract

This study investigates the optimization of microwave-assisted extraction (MAE) to recover ursolic acid (UA) from Hanfu apple pomace using response surface methodology (RSM). The effects of three key variables—extraction time, sample-to-solvent ratio, and ethanol concentration—on UA yield were studied. A Box-Behnken design was employed to model and optimize these variables. The results showed that extraction time had the most significant effect on UA yield, followed by ethanol concentration and sample-to-solvent ratio. The optimal conditions for UA extraction were 118.25 s for extraction time, a 1:30.86 sample-to-solvent ratio, and 82.23% ethanol concentration, with a predicted maximum UA yield of 89.92%. These results were validated with an actual UA yield of 88.87%, confirming the model's predictive reliability. The study highlights the efficiency of MAE for extracting UA, demonstrating its potential as a green extraction method for bioactive compounds from apple pomace. Further purification using XAD-7 resin improved UA purity significantly.

## 1 Introduction

Apple is one of the most popular fruits globally, consumed either fresh or as a processed product. A significant amount of the apples produced worldwide go to industrial processing to be converted into juice or cider. The 25%−30% of the weight of the original fresh apple remains as residual material after juice and cider production, which is comprised mainly of peels, seeds, leftover flesh (pulp), and stems, known as apple pomace. Apple pomace is an excellent source of valuable compounds, including dietary fibers, carbohydrates, triterpenoids such as ursolic acid, polyphenols, vitamins, amino acids, and minerals, which can be utilized as a low-cost source for the manufacture of high-value phytochemicals and bioactive compounds (1, 2). Among them, ursolic acid (UA; C30H48O3), which belongs to triterpenoids, has attracted specific attention due to its human health beneficial effects, having antioxidant and antibacterial activity, hepatoprotective and anticancer effects, immuno-modulating activity, and others (1). Various extraction methods have been used to obtain UA from apple pomace, including traditional methods such as maceration, soxhlet extraction, heat reflux extraction, and emerging green extraction techniques (1, 3). Traditional methods are often time-consuming, expensive, have low extraction yields, and require large volumes of organic solvents. Therefore, emerging green extraction techniques are being developed to increase extraction selectivity, minimize extraction time, and achieve better recovery rates. Several emerging green extraction methods have been used to extract phytochemicals and bioactive compounds from apple pomace, including ultrasound-assisted extraction, microwave-assisted extraction (MAE), accelerated solvent extraction, supercritical fluid extraction, enzyme-assisted extraction, high hydrostatic pressure processing, and pulsed electric field extraction. A higher efficiency of MAE over conventional extraction methods for UA extraction from *Lamii albi* has been reported (4). The MAE has also been successfully used for UA extraction from *gardenia* (5), loquat leaves (6), red jujubes (7), and cinquefoil herb (8), with a higher extraction yield, short processing time, higher efficiency, and lower solvent consumption. Nevertheless, several factors, including operational variables such as the type and volume of extraction solvents, solvent-to-sample ratio, microwave extraction time, and microwave power, influence the UA yields and rates, which need to be optimized. The effects of these factors can be either independent or interactive. To optimize UA extraction from apple pomace and determine the most favorable MAE conditions, the response surface methodology (RSM) can be implemented. RSM is a statistical method to optimize the extraction processes by generating a predictive mathematical model to explore the relationships between the variables and the response, allowing for the identification of optimal conditions. The primary benefit of RSM is that it requires fewer experimental trials than traditional optimization methods, thereby saving time, resources, and materials. Additionally, RSM provides valuable information about the significance of individual factors, their quadratic effects, and potential interactions between them (9, 10). In this study, a Box-Behnken Design (BBD), a type of RSM, was chosen to establish a mathematical model to predict UA extraction from apple pomace using MAE due to its efficiency and suitability for three-factor optimization, allowing the simultaneous evaluation of extraction time, sample-to-solvent ratio, and ethanol concentration on the UA yield. BBD is particularly effective because it avoids experiments at extreme conditions, reducing the likelihood of experimental failures while still effectively capturing the curvature of the response surface (10). The crude UA extract requires additional processing to achieve a final product with higher purity, typically involving steps such as adsorption, crystallization, centrifugation, washing, and drying of the raw material extract (4). Thus, the crude UA extract obtained from optimized MAE was further purified using XAD-7 resin by evaluating the sample loading flow rate, sample loading volume, and ethanol concentration, all of which affect purification efficiency.

## 2 Materials and methods

Hanfu apples cultivated in the Shenyang region (China) were used to obtain apple pomace. Apples were cut into quarters and blanched in hot water at 90°C for 2 min. The samples were then processed into juice in a small-scale plant or a juice extractor in the laboratory of Shenyang Agricultural University, and the residue remaining after juice separation, as apple pomace, was dried in a blast drying oven (Model: DHG-9036A, Shanghai Rongfeng Instrument Co., Ltd.) type at 40°C until constant weight. The dried samples were then powdered and sieved through 80-μm screen size to obtain a fine powder and stored at −20°C before UA extraction.

### 2.1 Experimental design and microwave-assisted extraction

Microwave-assisted extraction (MAE) of UA was performed using a Multifunctional Electric Flat Plate Microwave Oven (NN-GF361M, Shanghai Panasonic Microwave Oven Co., Ltd.) operating at 50 Hz with a maximum output power of 800 W. To determine the best range levels of the selected variables (60–180 s for extraction time; 1:10–1:50 for sample/solvent ratio; and 70%−90% for ethanol concentration), a single factor analysis method was used. Note that due to equipment limitations, a fixed power of 800 W was used for MAE in this study.

In the optimization of experimental factors, one variable was varied while keeping the other factors constant for each experiment, and all experiments were conducted three times.

Based on the single factor analysis results (see Section 3), three levels of each variable that influenced the UA recovery were as follows: 90, 120, and 150 s for extraction time; 1:20, 1:30, and 1:40 for sample/solvent ratio; and 75%, 80%, and 85% for ethanol concentration. The selected levels of independent variables were coded as −1, 0, and +1 and combined together to maximize the UA recovery from apple pomace with RSM.

To optimize the MAE process, Design Expert software (Version 8.0.6) was used to apply RSM-Box-Behnken Design (BBD) with three levels (maximum, minimum, and central) of each parameter to investigate the simultaneous effect of extraction time (A), sample/solvent ratio (B), and ethanol concentration (C) and their interaction effects on UA-MAE extraction from apple pomace and to determine the response pattern and establish a mathematical model to estimate UA extraction rate (level and yield) (Table 1). Seventeen experiments with three replications, including nine replications for the central points, were performed (Table 2), and the effect of independent variables with linear, quadratic, and interaction terms on the UA extraction (as response variable) was assessed by a quadratic polynomial regression model generated with the Design Expert software as follows:


$$\begin{array}{l} \text{Y}:\;\delta_{\text{0}}\text{+}\delta_{\text{1}}\text{A+}\delta_{\text{2}}\text{B+}\delta_{\text{3}}\text{C+}\delta_{\text{11}}{\text{A}}^{\text{2}}\text{+}\delta_{\text{22}}{\text{B}}^{\text{2}}\\ \text{ + }\delta_{\text{33}}{\text{C}}^{\text{2}}\text{+}\delta_{\text{12}}\text{AB+}\delta_{\text{23}}\text{BC+}\delta_{\text{13}}\;\text{AC} \end{array}$$

Y is estimated UA extraction amount, δ_0_ is a constant coefficient that fixed the response at the central point of the experiment, δ_1_, δ_2_, and δ_3_ are linear coefficients, δ_12_, δ_23_, and δ_13_ are interactive coefficients, and δ_11_, δ_22_, and δ_33_ are squared coefficients.

**Table 1:** The design of factors and levels used in the response surface experiment.

**Levels**	**Factors**		
	**Extraction time (A)**	**Sample/ solvent ratio (B)**	**Ethanol concentration (C)**
−1	90 s	1:20 g/mL	75%
0	120 s	1:30 g/mL	80%
1	150 s	1:40 g/mL	85%

**Table 2:** Box-Behnken experimental design matrix and predicted ursolic acid extraction.

**Runs**	**Extraction time (A)**	**Sample/solvent ratio (B)**	**Ethanol concentration (C)**	**Ursolic acid yield (%)**
1	−1	0	−1	76.96
2	0	0	0	89.62
3	0	0	0	87.98
4	0	1	1	76.36
5	1	0	−1	79.68
6	0	0	0	88.95
7	0	0	0	88.89
8	0	−1	1	77.39
9	0	−1	−1	79.56
10	−1	−1	0	76.02
11	0	0	0	88.54
12	1	−1	0	79.45
13	0	1	−1	78.05
14	−1	1	0	74.63
15	−1	0	1	73.54
16	1	1	0	78.78
17	1	0	1	77.44

The model was statistically analyzed, and the ANOVA was used to test the model adequacy and statistical significance of the regression coefficients, where *p* < 0.05 was considered statistically significant. The 2D contour graphs and 3D response surface plots were employed to study the interaction effects of independent variables on the response. The validity of the model for predicting the optimum response value was determined by comparing the average value of triplicate experiments under the optimal conditions and predicted values by the developed model.

### 2.2 Microwave-assisted extraction

The extraction was carried out under various MAE conditions according to the experimental design. Ten grams of dried apple pomace was weighed and placed into a 500-mL volumetric beaker, and different amounts of ethanol as the extraction solvent (1:20, 1:30, and 1:40 for solid/liquid ratio) at 75%, 80%, and 85% concentrations were added. The beakers containing samples were placed in the middle of the microwave oven over a rotating dish, and MAE was carried out for the selected extraction times (90, 120, and 150 s) using a Multifunctional Electric Flat Plate Microwave Oven (NN-GF361M, Shanghai Panasonic Microwave Oven Co., Ltd.) operating at 50 Hz with the maximum output power. The mixtures were then allowed to cool down to room temperature and filtered by using Whatman filter paper No. 1. The extraction solvents were removed under vacuum using a rotary evaporator (RV10 BASIC V-C, German IKA group) at 40°C, then lyophilized (−50°C, 0.1 mbar, 48 h), and the crude extracts of UA were stored at −20°C for UA analysis.

### 2.3 Determination of ursolic acid yield

The ursolic acid content of extracts was determined using a colorimetric method described by Chen et al. (11) and Murakami et al. (12) with some modifications. This method was chosen for the quantification of UA due to its simplicity, cost-effectiveness, and rapid execution during extraction optimization. The method is based on the reaction of oxidized ursolic acid by perchloric acid with vanillin with a maximum absorbance at 548 nm and distinctive purple color. The crude extracts were dissolved in 10 mL absolute ethanol, 1 mL of aliquots from the mixture were totally evaporated, and 0.2 mL of 5% vanillin-glacial acetic acid solution (w/v) and 0.8 mL perchloric acid were added. The mixtures were mixed well and incubated at 60°C for 15 min. The incubated samples were then cooled under running water for 2 min and made up to 10 mL with glacial acetic acid. The absorbance of the solutions was measured using a UV-Vis spectrophotometer at 548 nm. The concentrations of UA were calculated using a standard curve developed with the ursolic acid standard (Nanjing Yuanzhi Biological Technology Co., Ltd.) and were expressed as a percent of ursolic acid extraction using the following formula:


$$\text{Ursolic acid extraction rate }(\%)\text{=}\frac{\text{W2}}{\text{W1}}\times \text{100}\;$$

W1 is the total amount of ursolic acid in apple pomace samples before extraction; W2 is the total amount of ursolic acid in each sample extract.

### 2.4 Validation of vanillin-glacial acetic acid method

A standard curve was developed for pure ursolic acid using a colorimetric method described by Murakami et al. (12) and Chen et al. (11), taking 548 nm as the maximum absorbance for oxidized ursolic acid-vanillin complex. A stock solution of ursolic acid (10 mg/mL) was prepared in ethanol, and a calibration curve was established using serial dilutions ranging from 0.01 to 0.10 mg/mL. The results showed a regression of Y = 4.0393X+0.0059 and *R*^2^ = 0.9992 (Y is the absorbance at 548 nm and X is ursolic acid concentration as mg/mL), indicating a good linearity, which confirms the method proficiency for UA determination in the extracts.

### 2.5 Ursolic acid purification

#### 2.5.1 Preparation of XAD-7 resin

The purification of UA was performed only after extraction under the optimized condition to evaluate the effects of the purification step on the crude UA extract for potential industrial applications, using a commercial polymeric resin with an acrylic matrix, Amberlite XAD7 (particles size: 20–60 mesh, pore volume: 0.5 mL/g, specific surface area of 380 m^2^/g; Sigma-Aldrich, Merck, Darmstadt, Germany). The resin was prepared according to the supplier. In brief, the XAD-7 resin was transferred into a 3,000 mL beaker, and absolute ethanol was added to submerge the resin. After mixing well, the mixture was kept at ambient temperature for 24 h, and a 1.8 cm × 30 cm resin column was filled with the resin. The filled column was then rinsed with absolute ethanol at 2 BV/h until no turbidity was observed. The column was then rinsed with ultra-pure water at the same flow rate to wash ethanol. The column was soaked with 2 BV HCl 5%, flushed at 5 BV/h, washed with ultra-pure water at 5 BV/h, naturalized with NaOH 2%, and washed with ultra-pure water adjusting pH on 7. The prepared resin was then used for UA purification.

#### 2.5.2 Determination of dynamic adsorption-desorption conditions of XAD-7 resin

A 1.8 cm × 30 cm resin column was filled with the XAD-7 resin, and crude extracts dissolved in ethanol were loaded on the column using a constant flow pump. After equilibrium, impurities were washed with ultrapure water, and UA was eluted with ethanol. The effects of three independent variables, including loading flow rate (2–6 BV/h), sample loading volume (150–250 mL), and ethanol concentration (%) on UA purification, were evaluated. The adsorption and desorption rates of UA were calculated using the following formulas:


$$\begin{array}{l} \text{Adsorption rate }(\%)\;\text{=}\frac{{\text{C}}_{\text{0}}{\text{V}}_{\text{0}}\;-\;{\text{C}}_{\text{1}}{\text{V}}_{\text{1}}}{{\text{C}}_{\text{0}}{\text{V}}_{\text{0}}}\times \text{100}\\ \text{ Desorption rate }(\%)\text{=}\;\frac{{\text{C}}_{\text{2}}{\text{V}}_{\text{2}}}{{\text{C}}_{\text{0}}{\text{V}}_{\text{0}}\;-\;{\text{C}}_{\text{1}}{\text{V}}_{\text{1}}}\times \text{100} \end{array}$$

Where C_0_ is the ursolic acid concentration in the sample solution (mg/mL), V_0_ is the sample volume (mL), C_1_ is the ursolic acid concentration in the adsorption solution (mg/mL), V_1_ is the volume of adsorption solution (mL), C_2_ is the ursolic acid concentration in the eluent (mg/mL), and V_2_ is the volume of eluent (mL).

The purity of extracts was then determined by high performance liquid chromatography (HPLC) (Nexera UHPLC LC-30A) using a C18 reverse phase column. The mobile phase was acetonitrile: 0.3% of phosphoric acid water (90:10), column temperature of 30°C, gradient elution (0–35 min, 25–60), injection volume of 10 μL, detection wavelength of 210 nm, and flow rate of 1.0 mL/min.

### 2.6 Statistics

Statistical analyses of the data were performed using SPSS software (version 13.0 for Windows, SPSS, Inc., Chicago, IL, USA) by comparing the means of three independent experiments with one-way ANOVA followed by Duncan's Multiple Range test for comparing the differences between mean values at a significance level of *p* < 0.05. Results were expressed as mean value ± standard deviation.

## 3 Results and discussion

### 3.1 Single factor analysis

#### 3.1.1 Extraction time

**Figure 1:**
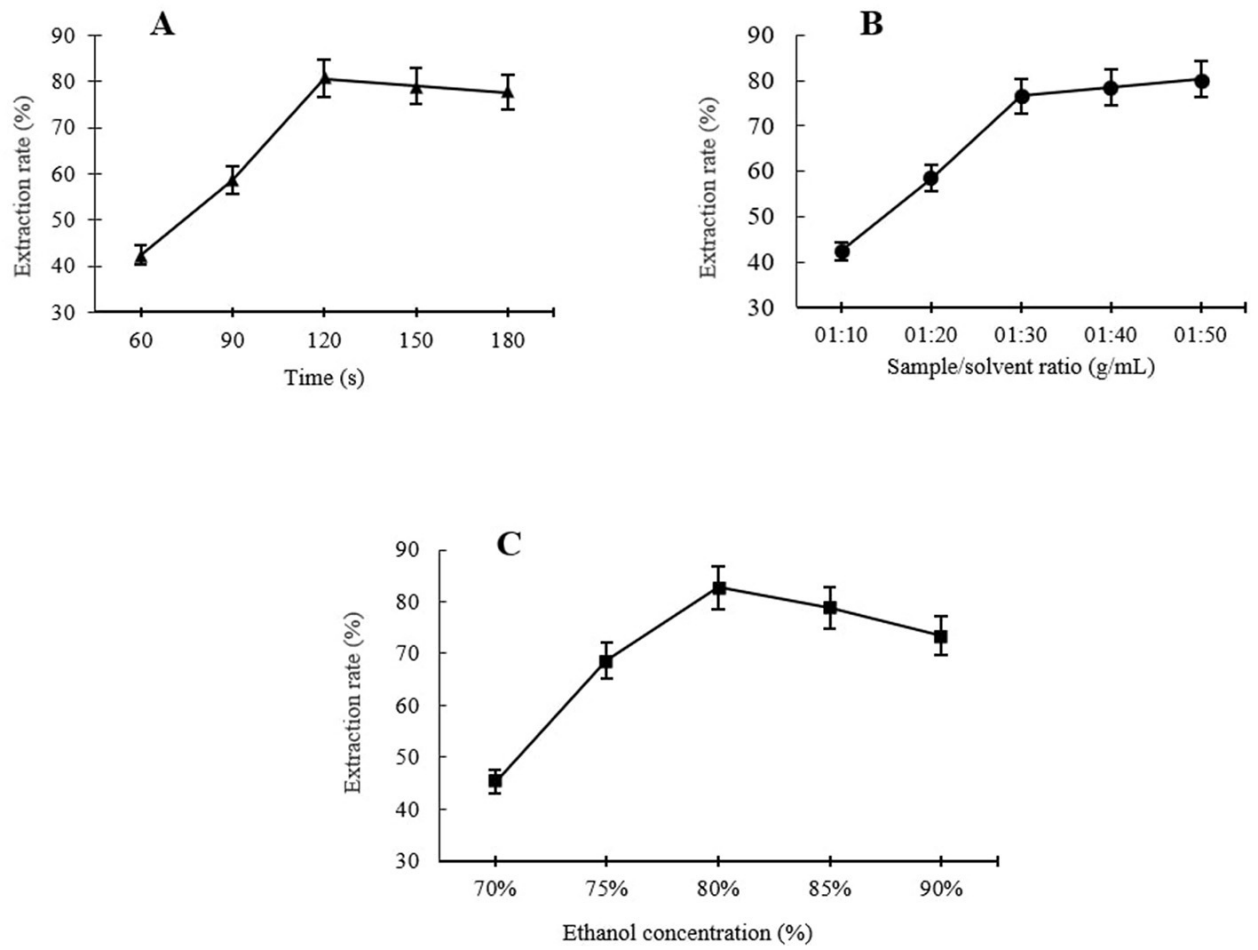
Single factor analysis of different levels of extraction times **(A)**, sample to solvent ratio **(B)**, and ethanol concentration **(C)** on MAE of UA from Hanfu apple pomance.

Using a fixed ethanol concentration of 80% and a fixed sample-to-solvent ratio of 1:30, the effects of different extraction times, including 60, 90, 120, 150, and 180 s, on ursolic acid MAE rates were evaluated. As shown in Figure 1A, the yield of UA raised at first with increasing treatment time from 60 s and reached a maximum extraction yield of 80.59% at 120 s. The increase in the UA extraction rate with longer extraction times may be due to greater disruption of the plant cell wall and matrix, which enhances UA release from the samples and accelerates its diffusion into the extraction solvent (13). However, further increases in extraction time, i.e., 150 and 180 s, resulted in a slight decrease in UA extraction, probably because of UA decomposition under excessively lengthening extraction time due to overexposure to microwave radiation (13, 14). Similar results were found by Xiao et al. (13), who observed a similar extraction behavior of UA from *Hedyotis diffusa* at different MAE times. Verma et al. (14) also observed a decrease in UA-MAE following the extension of the treatment time, i.e., the UA extraction increased under microwave treatment for up to 5 min, while after 6 min, it started to decrease. Therefore, the extraction times were selected as 90, 120, and 150 s for the RSM optimization study.

#### 3.1.2 Sample-to-solvent ratio

One of the considerable advantages of using MAE is that this extraction method consumes less solvent for a high extraction recovery compared to conventional extraction methods (15). Using the MAE technique, the sample-to-solvent ratio is a key factor influencing the yield of bioactive compounds extracted from apple pomace, as demonstrated in several previous studies. In Figure 1B, it can be seen that using a fixed ethanol concentration of 80% and extraction time of 120 s, at the beginning, the UA yield significantly increased in a certain sample-to-solvent ratio range; UA extraction increased sharply with the ratio increase from 1:20 to 1:30, and after that, a further increase in the sample-to-solvent ratio, i.e., 1:40 and 1:50 ratios, did not provide a significant improvement in the extraction efficiency. According to Xiao et al. (13), at low to moderate levels of sample-to-solvent ratio, increasing the ratio promotes a concentration gradient, resulting in an increased diffusion rate, which leads to a higher extraction level by solvent. In addition, the level of cell wall disruption in the apple pomace increases with increasing the solvent ratio to a certain level, leading to higher leaching out rates. This can be explained by the fact that a larger volume of solvent, up to a certain level, causes excessive swelling of the materials (16) and allows the microwaves to be absorbed directly by the materials. A larger sample-to-solvent ratio did not yield further improvements in extraction efficiency. This suggests that the available extractable UA within the plant matrix had already been fully extracted. Similar results were reported for UA extraction from *Hedyotis diffusa* and *Eucalyptus* × *hybrida Maiden* using MAE by Xiao et al. (13) and Verma et al. (14), respectively. Hence, 1:20, 1:30, and 1:40 were selected as suitable levels of sample-to-solvent ratios for RSM.

#### 3.1.3 Ethanol concentration

The efficiency of MAE is significantly affected by solvent selection; specifically, the solvent's dielectric constant and dissipation factor influence extraction yield (17). For this study, an aqueous ethanol solution was selected as the extraction solvent due to its established efficacy in extracting bioactive compounds from a range of plant-based samples, in addition to its accessibility and low toxicity (18). The trends of the UA extraction under different ethanol concentrations (70%−90%) at a constant extraction time of 120 s and 1:30 sample-to-solvent ratio are shown in Figure 1C. The UA level of the extracts showed an increase with increasing the ethanol concentration at a range between 70% and 80% (v/v), with the maximum yield reaching 80% ethanol concentration of 82.68% UA extraction. The extraction rate then fell with further increase of ethanol concentration, i.e., the UA extraction for 85% and 90% ethanol concentration were 78.82% and 73.45%, respectively. In agreement with these results, Araújo et al. (19) reported similar influencing trends of ethanol concentration on microwave-assisted extraction of phenolic compounds from avocado seeds. Similar results were also reported by Luo et al. (18), who reported a continuous increase in microwave-assisted extraction of FRAP, TPC, and TEAC from *Akebia trifoliata* peels between 20% and 50% (v/v) of ethanol concentration with a gradual decrease in extraction rate by a further increase in ethanol concentration. This trend may be attributed to the enhanced solubility and diffusivity of phenolic compounds as the dielectric constant of the solvent decreases with increasing ethanol concentration. However, at high ethanol concentrations, i.e., close to 100%, the highly pure solvent can dehydrate plant tissues and cause protein denaturation, ultimately leading to a reduction in extraction yield (20). Therefore, 75%, 80%, and 85% ethanol concentrations were chosen for RSM optimization of UA extraction from Hanfu apple pomace.

### 3.2 Experimental design and statistical analysis

#### 3.2.1 Fitting the model

The experimental results of a response surface for UA extraction from apple pomace using three factors, including extraction time, sample-to-solvent ratio, and ethanol concentration, each at three levels using BBD, are shown in Table 2. With analysis of variances (ANOVA), a second-order polynomial model was developed to predict the UA extraction yields from apple pomace to show the relationship between the UA extraction rates and the independent variables as follows:


$$\begin{array}{l} \text{Y= 88}.\text{80 + 1}.\text{78A }-\text{ 0}.\text{57B }-\text{ 1}.\text{19C + 0}.\text{18AB + 0}.\text{29AC}\\ \text{ + 0}.\text{12BC }-\text{ 6}.{\text{26A}}^{\text{2}}\;-\text{ 5}.{\text{32B}}^{\text{2}}\;-\text{ 5}.{\text{64C}}^{\text{2}} \end{array}$$

The ANOVA was used to establish the statistical significance of model terms, i.e., linear, quadratic, and interaction coefficients effects of the variables on the UA extraction as the response of the model (Table 3). The goodness of fit of the constructed model was evaluated by determination coefficient (R^2^) and adjusted determination coefficient (adjusted R^2^). The R^2^ value depends on the number of model terms and increases only when the model promotes to higher degrees with additional terms being added to the model. Therefore, adjusting R^2^, which accounts for the number of terms in the fitted model, is necessary for a more accurate assessment of the model's goodness-of-fit. A closer value of these coefficients to 1 and each other indicates a suitable model to predict the experimental values. The high *R*^2^ value of 0.9961 and adjusted *R*^2^ of 0.9910 indicate a strong correlation between the experimental and predicted values, signifying that the models are reliable. Indeed, based on R^2^ and adjusted R^2^ values, only <1% of the total variations cannot be explained by the developed model. The model's high *F-value* of 197.66 and low *p-value* of <0.0001 demonstrate the model's significance, indicating that the variation in the response can be explained by the regression equation. The model's validity and suitability were further confirmed by an insignificant lack of fit, with an *F-value* of 0.51 and a *p-value* of 0.6981, indicating a good fit. This suggests that the predicted model reasonably represents the observed values, sufficiently explaining the response. Thus, the model developed in this study can be used to predict the MAE rate of UA from Hanfu apple pomace. It is worth mentioning that, according to the *F-values*, the extraction time has the most effect on UA extraction, followed by ethanol concentration and sample-to-solvent ratio, respectively.

**Table 3:** Analysis of variance for regression model of ursolic acid extraction from apple pomace.

**Source**	**Sum of squares**	**Degree of freedom**	**Mean square**	***F-value***	***p-value***	**Prob > F**
Model	506.21	9	56.25	197.66	<0.0001	Significant
A- Extraction time	25.21	1	25.21	88.58	<0.0001	
B- Sample/solvent ratio	2.64	1	2.64	9.3	0.0186	
C- Ethanol concentration	11.33	1	11.33	39.81	0.0004	
AB	0.13	1	0.13	0.46	0.5214	
AC	0.35	1	0.35	1.22	0.3053	
BC	0.058	1	0.058	0.2	0.6664	
A^2^	164.76	1	164.76	579.01	<0.0001	
B^2^	119.19	1	119.19	418.86	<0.0001	
C^2^	133.72	1	133.72	469.92	<0.0001	
Residual	1.99	7	0.28			
Lack of fit	0.55	3	0.18	0.51	0.6981	Not significant
Pure error	1.44	4	0.36			
Cor total	0.048	16				

### 3.3 Analysis of response surface of various factors on UA extraction rate

**Figure 2:**
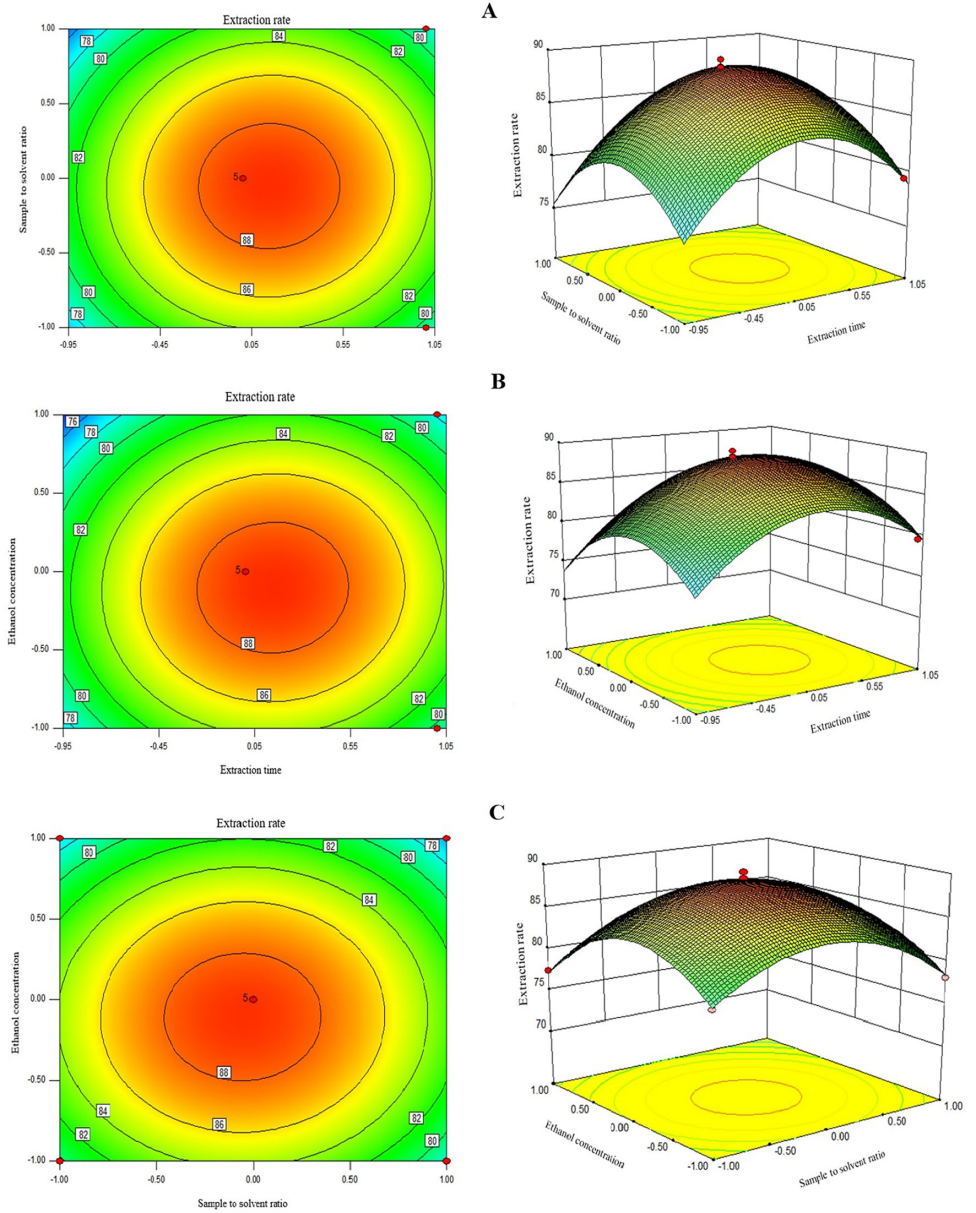
The 2D and 3D repsonse plots showing the interactive effects of extraction times and sample to solvent ratio **(A)**, ethanol concentration and extraction times **(B)**, and sample solvent ration and ethanol concentration **(C)** on the UA extraction time.

Two-dimensional contour graphs and three-dimensional surface plots were used to visualize the interactive effects of two factors on the UA extraction as the response at the time while keeping the other factor at level zero (Figure 2). The response surface plot in Figure 2A illustrates the interactive effects of extraction time and sample/solvent ratio on the UA extraction rate. As can be seen, the UA extraction rate enhanced with a rise in both parameters, and the maximum UA yield was achieved when these factors were in their central points, i.e., 120 s and 1:30 sample/solvent ratio, respectively. The elliptical shape of the 3D plot in Figure 2A indicates that increasing both extraction time and sample/solvent ratio first increased and then decreased the response, which explains why the quadratic coefficients of these factors were negative. A decrease in UA extraction rate at a long extraction time can be explained by UA degradation due to overexposure to microwave radiation at an extended time beyond the optimum point (13, 14). A similar decreasing pattern in the extraction of TPC (21) and polyphenols (22) from apple pomace using MAE with an increasing sample/solvent ratio has also been reported by others. This could be due to the maximum extraction of extractable UA from the apple pomace, where further increases in the solvent-sample ratio do not improve extraction efficiency (22).

As depicted in Figure 2A, round contour lines of 2D contour graph indicate non-significant interaction effects of extraction time and sample/solvent ratio on UA extraction efficiency. This is evidenced by a higher *p-value* than the statistically significant level of 0.05 in this study; i.e., *p-value* for the interaction term was 0.52 (Table 3). Similarly, extraction time and solvent concentration showed a non-significant interaction effect in the model with a *p-value* of 0.30 and round contour lines of 2D contour graph (Figure 2B). Non-significant interaction terms in the model indicate the possibility of interpreting the individual effect of independent factors as their impact is not affected by the level of the other factors. Figure 2B shows the 3D surface plot of the combined effects of extraction time and solvent concentration on UA extraction efficiency from Hanfu apple pomace. The UA yield first increased with an increase in solvent concentration to the highest UA extraction of ≈88% at 80% of ethanol concentration and then leveled off at higher ethanol concentrations. Several authors have encountered an optimal ethanol concentration for extracting phenolic compounds from plant-based materials (23). This can be explained by polarity mismatch of the solvent with the bioactive compound, where it might no longer be compatible with the target compounds, which are typically extracted best using solvents that match their polarity, such as aqueous-organic mixtures (e.g., water and ethanol) (19). Negative quadratic coefficients of sample/solvent ratio and solvent concentration factors in the model regression equation and an elliptical shape of a 3D plot of these factors (Figures 2B, C) also indicate that increasing these variables first increases the response to reach a maximum and then decreased the UA extraction rate. Similar to the other two factors, a 2D contour graph and a *p-value* of 0.66 show a non-significant interactive effect of sample/solvent ratio and solvent concentration variables on UA extraction. It is also worth mentioning that the slope of the curved surfaces of the 3D plots also confirms, in agreement with F-values, that in order of time, ethanol concentration and sample-to-solvent ratio have the greatest effect on UA extraction from apple pomace, respectively.

### 3.4 Determination of optimum condition and verification of predictive model

The optimal microwave-assisted extraction parameters were determined by the RSM optimization in Design-Expert software using the desirability function approach, which evaluates a point that maximizes the response. The optimum extraction condition obtained from Design-Expert software was found to be an extraction time of 118.25 s, a sample/solvent ratio of 1:30.86, and a solvent concentration of 82.23%, with a predicted maximum UA yield of 89.92%. This optimal condition was then used to validate the experimental and predicted yield of the UA extraction using the model equation. An average UA extraction amount of 88.87% was obtained from triplicate experiments under the optimized condition with a standard deviation of 1.44%. Statistical analysis of the results showed no significant difference (*p* > 0.05) between the predicted extraction yield of ursolic acid and experimental values that indicated a high predictive ability of the mathematical model for the UA extraction from Hanfu apple pomace.

### 3.5 XAD-7 resin purification

The crude solvent extracts from plant-based materials typically require further processing to obtain target products with higher purity. Macroporous resins are widely used for the purification of crude bioactive extracts of plant-based materials due to their high efficiency, low cost, ease of manufacturing, and simple operation. XAD-7, a polymeric resin with acrylic matrix, is a moderately polar XAD resin used to adsorb relatively polar compounds up to MW 60,000 from non-aqueous solvents in a pH range of 0–14 (24). In this study, the effects of loading flow rate and sample loading volume on adsorption rate and ethanol concentration on desorption rate were evaluated.

**Figure 3:**
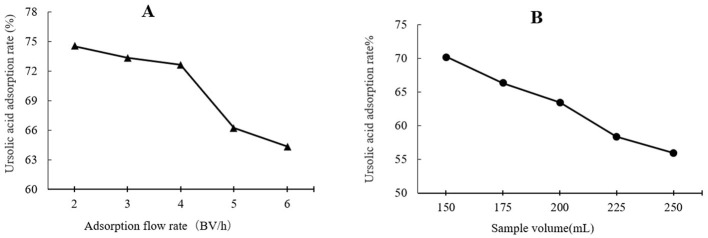
Effects of flow rate **(A)** and sample volume **(B)** on UA adsorption on XAD-7 resin.

**Figure 4:**
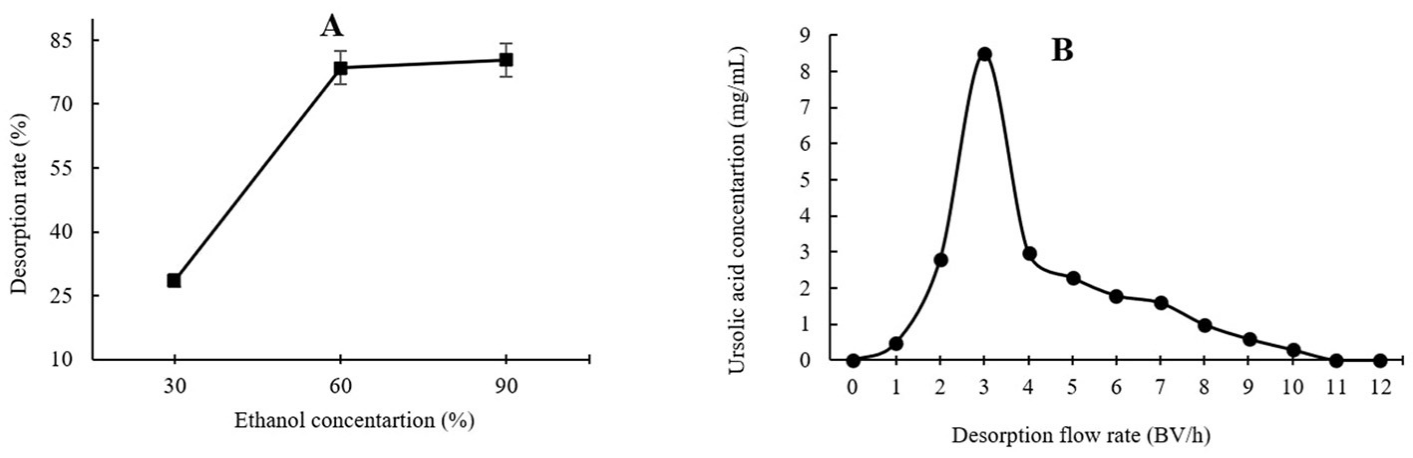
Effects of ethanol concentration **(A)** and desorption flow rate **(B)** on the resin desorption performance.

**Figure 5:**
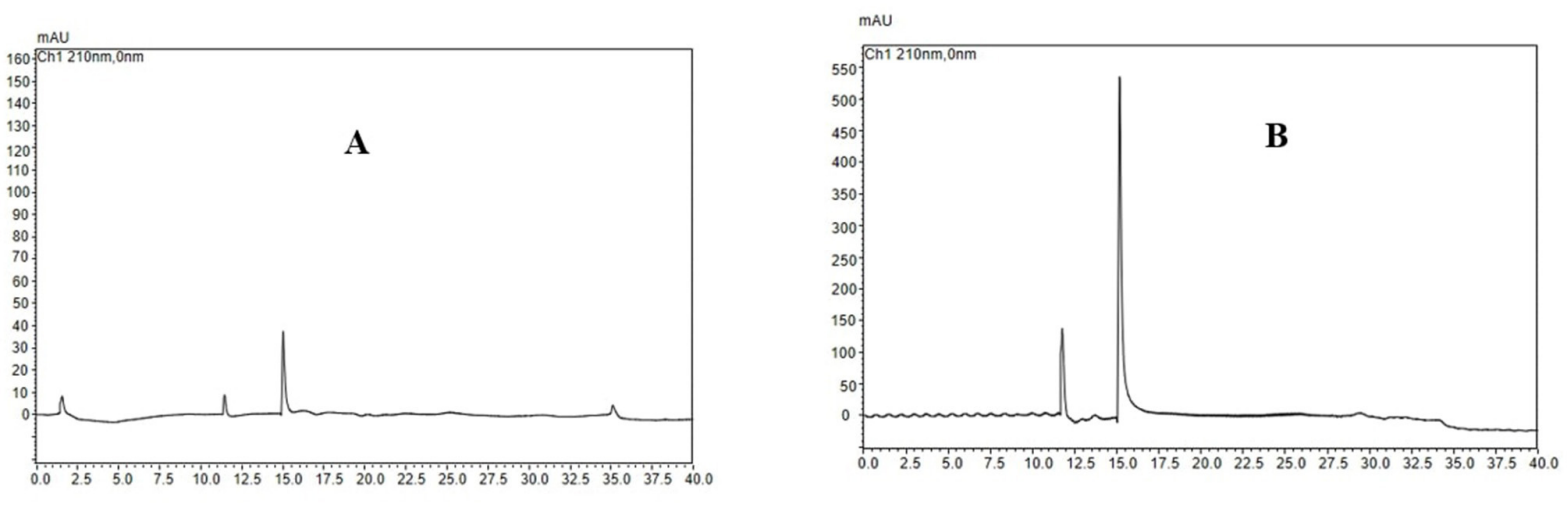
HPLC chromatogram of extracted ursolic acid before **(A)** and after **(B)** XAD-7 resin purification.

As shown in Figure 3A, the highest UA adsorption rate of 74.54% on the XAD-7 resin was recorded for 2 BV/h of sample loading flow rate with a slight but not significant decrease for higher flow rates of 3 BV/h and 4 BV/h. Further increases in sample loading flow rate, i.e., at 5 BV/h and 6 BV/h, led to a significant (*p* < 0.05) decrease in UA adsorption rate. In line with these findings, 2022 (Section 3.2) also reported that increasing the sample loading flow rate negatively affected the dynamic adsorption of polyphenols on XAD-16 resin. Similarly, Xi et al. (25) observed the same trend for polyphenol adsorption from sweet potato leaves using AB-8 resin, which, like XAD-7, is slightly polar. Therefore, a slower sample loading flow rate would improve the adsorption rate of the target molecules, where a longer time provides enough time for these compounds in the eluent to interact with active sites on the resin surface (26). Thus, based on the results, a sample loading flow rate of 4 BV/h can be the best choice for UA crude extract from apple pomace adsorption on XAD-7 resin. Figure 3B shows the effects of sample loading volume in a 150 mL – 250 mL range on the adsorption rate. With increasing the sample loading volume at a fixed concentration, the adsorption rate gradually decreased, i.e., the adsorption rates were 70.24% and 55.95% for sample volumes of 150 mL and 250 mL, respectively. This can be explained by an over sample loading, greater than the XAD-7 resin capacity where either the resin could be saturated, and thus, the excessive solute cannot be held by absorbent or due to not enough time for the completion of adsorption of a large amount of eluent to interact with the active sites on the resin surface (26). Figure 4A shows the effects of ethanol concentration on the UA desorption rate from the XAD-7 resin. For an ethanol concentration of 30%, the desorption rate was 28.58% and increased to 78.58% when the ethanol concentration increased to 60%. A further increase in the ethanol concentration to 90%, however, did not result in a higher desorption rate. Similar results were reported by Wang et al. (27), who found that 60% of ethanol was the optimum solvent concentration for maximum desorption of polyphenols from D101 resin. Interacted molecules are released from resin due to competing forces: the attraction between these molecules and the resin and their tendency to dissolve in the solvent. When the attraction to the resin weakens, adsorbed molecules are more likely to dissolve into the solvent. The highest desorption rate, seen with a 60% ethanol solution, is likely because the polarity of the ethanol best matches that of the UA, making it easier for them to detach from the resin (27). Using a 60% ethanol concentration, the effects of different desorption flow rates were evaluated (Figure 4B). The elution peak was between 2 and 4 BV/h, and using 11 BV/h, almost all the adsorbed UA was eluted. Comparing HPLC chromatograms of unpurified and purified UA proves the efficiency of XAD-7 macroporous resin in increasing the purity of UA extracts (Figure 5).

## 4 Conclusions

This study successfully optimized the microwave-assisted extraction (MAE) of ursolic acid (UA) from Hanfu apple pomace using response surface methodology (RSM). The investigation revealed that extraction time had the most significant influence on UA yield, followed by ethanol concentration and sample-to-solvent ratio. The optimal extraction conditions were identified as 118.25 s for extraction time, a 1:30.86 sample-to-solvent ratio, and 82.23% ethanol concentration, which led to a maximum predicted UA yield of 89.92%. Validation experiments confirmed the accuracy of the model, yielding 88.87% UA under optimal conditions. One limitation of the study is that only one microwave power setting (800 W) was evaluated; future research could investigate the effect of varying microwave power on extraction efficiency.

Additionally, the purification of the crude extract using XAD-7 resin further enhanced the UA purity, demonstrating the resin's effectiveness in processing bioactive compounds. This study demonstrates that MAE is an efficient, rapid, and environmentally friendly method for extracting valuable bioactive compounds such as UA from apple pomace, supporting its potential for industrial applications in the production of phytochemicals. Future studies could focus on scaling up the process and exploring the economic feasibility of implementing MAE for large-scale operations.

## Data Availability

The original contributions presented in the study are included in the article/supplementary material, further inquiries can be directed to the corresponding author.
